# Releasing the Cortical Brake by Non-Invasive Electromagnetic Stimulation? rTMS Induces LTD of GABAergic Neurotransmission

**DOI:** 10.3389/fncir.2016.00096

**Published:** 2016-11-28

**Authors:** Maximilian Lenz, Andreas Vlachos

**Affiliations:** Institute of Anatomy II, Faculty of Medicine, Heinrich-Heine-University DüsseldorfDüsseldorf, Germany

**Keywords:** GABA_A_-receptors, gephyrin, parvalbumin, GAD65, somatic inhibition, dendritic inhibition

## Abstract

Repetitive Transcranial Magnetic Stimulation (rTMS) is a non-invasive brain stimulation technique which modulates cortical excitability beyond the stimulation period. However, despite its clinical use rTMS-based therapies which prevent or reduce disabilities in a functionally significant and sustained manner are scarce. It remains unclear how rTMS-mediated changes in cortical excitability, which are not task- or input-specific, exert beneficial effects in some healthy subjects and patients. While experimental evidence exists that repetitive magnetic stimulation (rMS) is linked to the induction of long-term potentiation (LTP) of excitatory neurotransmission, less attention has been dedicated to rTMS-induced structural, functional and molecular adaptations at inhibitory synapses. In this review article we provide a concise overview on basic neuroscience research, which reveals an important role of local disinhibitory networks in promoting associative learning and memory. These studies suggest that a reduction in inhibitory neurotransmission facilitates the expression of associative plasticity in cortical networks under physiological conditions. Hence, it is interesting to speculate that rTMS may act by decreasing GABAergic neurotransmission onto cortical principal neurons. Indeed, evidence has been provided that rTMS is capable of modulating inhibitory networks. Consistent with this suggestion recent basic science work discloses that a 10 Hz rTMS protocol reduces GABAergic synaptic strength on principal neurons. These findings support a model in which rTMS-induced long-term depression (LTD) of GABAergic synaptic strength mediates changes in excitation/inhibition-balance of cortical networks, which may in turn facilitate (or restore) the ability of stimulated networks to express input- and task-specific associative synaptic plasticity.

## Introduction

A remarkable property of the central nervous system is its ability to respond to specific stimuli with lasting structural, functional and molecular adaptations. Such “long-lasting (or permanent) transformations arising in particular systems of neurons as a result of appropriate stimuli” have been coined the term *plasticity* by Konorski (1948). In 1949 Donald Hebb’s famous work on associative plasticity was published (Hebb, 1949), which was experimentally proven ~25 years later by Bliss and Lomo (1973), who carried out the first *in vivo* long-term potentiation (LTP) experiments at excitatory entorhino-hippocampal projections of anesthetized rabbits. Since that time, various experimental LTP- and long-term depression (LTD)-paradigms were used to assess and better understand the cellular and molecular mechanisms of input-specific associative synaptic plasticity and its relevance for physiological brain function (for reviews see e.g., Bliss and Collingridge, 1993; Nicoll and Malenka, 1999; Huganir and Nicoll, 2013; Nicoll and Roche, 2013).

Years later, evidence for human cortical plasticity came from studies employing Transcranial Magnetic Stimulation (TMS; Barker et al., 1985; reviewed in Ziemann et al., 2008). TMS is a non-invasive brain stimulation technique currently used in clinical practice for diagnostic and therapeutic purposes, e.g., for the treatment of mood-disorders (Lefaucheur et al., 2014). Based on the physical principle of electromagnetic induction, TMS is capable of depolarizing cortical neurons through the intact scalp and skull of awake, non-anesthetized subjects (Barker et al., 1985). When applied repeatedly a long-lasting modulation of cortical excitability is observed, which outlasts the stimulation period and is detectable minutes to hours after stimulation (e.g., Stefan et al., 2000, 2002; Wolters et al., 2003). Even though a considerable degree of inter- and intra-individual variability has been reported (e.g., Müller-Dahlhaus et al., 2008; Hamada et al., 2013; Goldsworthy et al., 2014; López-Alonso et al., 2014; Nettekoven et al., 2015), pharmacological approaches and analogies to basic research findings have indicated that repetitive TMS (rTMS) modulates cortical excitability via “LTP-like” or “LTD-like” excitatory synaptic mechanisms (Ziemann et al., 2008). Indeed, studies employing animal models of rTMS (both *in vitro* and *in vivo*) disclose that rTMS is capable of inducing long-lasting changes of glutamatergic neurotransmission onto principal neurons (Levkovitz et al., 1999; Tokay et al., 2009; Gersner et al., 2011; Ghiglieri et al., 2012; Vlachos et al., 2012; Ma et al., 2013; Sykes et al., 2013; Volz et al., 2013; Lenz et al., 2015; Tang et al., 2015). Seemingly consistent with these observations, it was shown for example that rTMS modulates tactile learning performance in healthy animals (Mix et al., 2010), improves spatial learning in experimental vascular dementia (Wang et al., 2015), and promotes motor learning after stroke in humans in some situations (Brodie et al., 2014). However, it remains unclear how rTMS-induced associative plasticity, i.e., LTP or LTD of excitatory synapses, which is induced by trains of external electromagnetic pulses that are not task- or input-specific, can exert positive effects on motor and cognitive function under physiological and pathological conditions (see also Ridding and Rothwell, 2007).

Considering recent basic research findings, which reveal an important role of *local disinhibitory networks* in promoting associative learning and memory (e.g., Letzkus et al., 2011, 2015; Pi et al., 2013; Wolff et al., 2014; Fu et al., 2015), in this concise review article we aim at putting forward the idea that rTMS may affect cortical plasticity by modulating local inhibitory networks. In fact, an rTMS-induced increase in cortical excitability may not only reflect LTP of excitatory synapses, but could also reside in LTD of GABAergic neurotransmission, i.e., a reduction in inhibitory synaptic strength (see also Lee and Maguire, 2013; Wang and Maffei, 2014). Thus, rTMS may assert its positive effects by dampening GABAergic neurotransmission onto principal neurons, which may facilitate the ability of cortical networks to express task- and input-specific associative plasticity of excitatory synapses.

## Role of Disinhibitory Networks in Behavioral Learning

Despite a wealth of information on the cellular and molecular mechanisms underlying associative synaptic plasticity (Huganir and Nicoll, 2013), it remains not well understood how learning and memory formation are implemented at the network level. Recent studies employing modern optogenetic approaches in behaving animals (e.g., Ciocchi et al., 2010; Letzkus et al., 2011; Wolff et al., 2014) have brought an interesting concept back to our attention, which was suggested ~50 years ago by Young (1964). Based on the observation that inhibitory interneurons control the activity and excitability of cortical principal neurons (for a recent review on GABAergic interneurons, see Tremblay et al., 2016), it has been proposed that a transient reduction in inhibitory neurotransmission, i.e., *disinhibition*, could prime the ability of cortical networks for the subsequent expression of input-specific associative plasticity. Indeed, a series of animal studies support the notion that decreased inhibition improves learning (Collinson et al., 2002, 2006; Botta et al., 2015), while increased GABAergic neurotransmission impairs learning and memory formation (Davis, 1979; Sanger and Joly, 1985; McNaughton and Morris, 1987; Brioni et al., 1989; Arolfo and Brioni, 1991; Harris and Westbrook, 1995). Meanwhile, (opto-)genetic studies have started deciphering the functional connectivity of disinhibitory circuits and their relevance in associative synaptic plasticity and behavioral learning (reviewed in Letzkus et al., 2015), thus also confirming David Marr’s proposal (Marr, 1971) that local inhibitory networks set the threshold for producing long-term excitatory synaptic modifications (see also Douglas et al., 1982; Hsu et al., 1999; Ormond and Woodin, 2011; Bachtiar and Stagg, 2014).

**Figure 1:**
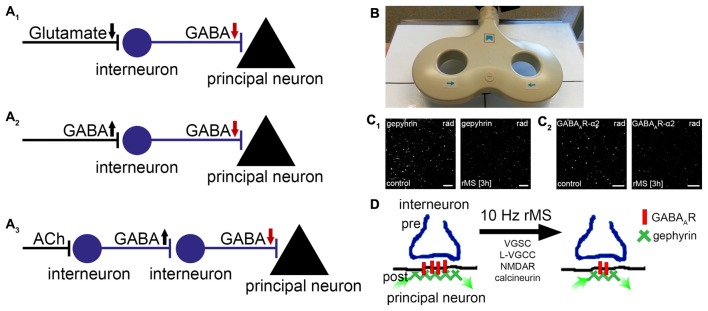
**Repetitive magnetic stimulation (rMS) induces long-term depression (LTD) of inhibitory postsynapses on principal neurons. (A_1–3_)** Local disinhibitory networks have been recently implicated in gating the ability of principal neurons to express associative excitatory synaptic plasticity. Schematic representation of mechanisms mediating disinhibition of principal neurons (gamma-aminobutyric acid, GABA; acetylcholine, ACh; arrows indicate direction of changes in neurotransmission). **(B)** Picture depicting a figure-of-eight magnetic stimulation coil (70 mm outer wing diameter). **(C)** Recent basic science work discloses that 10 Hz rMS induces Ca^2+^-dependent LTD of inhibitory postsynapses on principal neurons. These changes depend on the remodeling of gephyrin, the major postsynaptic scaffolding protein to which GABA_A_-receptors anchor. **(C_1,2_)** show examples of original data illustrating changes in gephyrin and GABA_A_-receptor subunit α2 clusters 3 h after 10 Hz rMS of organotypic slice cultures (CA1 stratum radiatum, rad; scale bars: 4 μm; see, Lenz et al., 2016). **(D)** Activation of voltage-gated sodium channels (VGSC), L-type voltage-gated calcium channels (L-VGCC), *N*-methyl-D-aspartate receptors (NMDAR), and calcineurin protein phosphatase is required during stimulation for rMS-induced LTD of inhibition to occur. It is conceivable that rTMS-induced local disinhibition may facilitate the ability of stimulated neurons to express task-/input-specific associative excitatory synaptic plasticity.

It appears to be well-accepted that *local disinhibition* can be achieved through plasticity of inhibitory synapses (Froemke, 2015) and by a set of distinct network mechanisms (Figure 1A), e.g.: (1) changes in glutamatergic drive onto local inhibitory interneurons; (2) modulation of long-range inhibitory projections innervating local inhibitory neurons; or (3) neuromodulators activating local inhibitory neurons, which in turn inhibit local interneurons projecting onto principal neurons (for a comprehensive review, see Letzkus et al., 2015; see also Griffen and Maffei, 2014; Caroni, 2015; Froemke, 2015). From a clinical point of view, these findings make local disinhibitory networks attractive targets for therapeutic intervention, considering that alterations in inhibitory synaptic plasticity and excitation/inhibition-balance have been linked to behavioral and cognitive dysfunction in various brain diseases (Steinberg et al., 2015), such as schizophrenia (Yizhar et al., 2011; Rowland et al., 2013), autism (Rubenstein and Merzenich, 2003; Rojas et al., 2014) or panic disorders (Long et al., 2013).

## rTMS-Effects on Inhibitory Neurotransmission

While conclusive experimental evidence exists that rTMS affects glutamatergic neurotransmission (e.g., Levkovitz et al., 1999; Tokay et al., 2009; Vlachos et al., 2012; Lenz et al., 2015), structural, functional and molecular adaptations of inhibitory synapses in response to rTMS remain not well understood. Nevertheless, basic science work from recent years has demonstrated that activity markers and calcium binding proteins of inhibitory interneurons are modulated by rTMS (Trippe et al., 2009; Funke and Benali, 2011; Volz et al., 2013; Labedi et al., 2014; Mix et al., 2015). For example, intermittent theta burst stimulation reduces parvalbumin (PV)-expression in fast-spiking interneurons of the rat cortex, while continuous theta burst stimulation and 1 Hz rTMS predominantly affect calbindin-expression (Trippe et al., 2009; Benali et al., 2011; Volz et al., 2013). As PV-expressing interneurons primarily affect somatic inhibition, while calbindin-expressing interneurons mediate dendritic inhibition (Mátyás et al., 2004), these findings imply that distinct rTMS-protocols may affect specific aspects of inhibition and therefore network activity and function (Mix et al., 2010; Funke and Benali, 2011; Volz et al., 2013). However, the assessment of histochemical markers or changes in gene expression (including work on the effects of rTMS on glutamate decarboxylase (GAD)-expression, which catalyzes the decarboxylation of glutamate to GABA; Volz et al., 2013) provide only indirect measures for actual changes in GABAergic neurotransmission. It has thus remained unclear whether rTMS is indeed capable of inducing long-lasting changes in inhibitory neurotransmission.

In a recent study after-effects of electromagnetic stimulation on GABAergic neurotransmission were studied (Lenz et al., 2016). Using a combination of structural, functional and molecular techniques in organotypic hippocampal slice cultures, which were stimulated with a conventional figure-of-eight coil (70 mm outer wing diameter; Figure 1B), we obtained evidence that a 10 Hz stimulation protocol induces Ca^2+^-dependent LTD of inhibitory synapses on principal neurons. The functional changes were accompanied by a remodeling of gephyrin, the major postsynaptic scaffolding protein to which GABA_A_-receptors are anchored (Kneussel and Betz, 2000; Kneussel and Loebrich, 2007; Tyagarajan and Fritschy, 2014). While gephyrin-mRNA and protein levels were not affected, the size and stability of gephyrin-aggregates were markedly reduced after rTMS (Figure 1C). These posttranslational effects of rTMS required the activation of voltage-gated sodium channels (VGSC), *N*-methyl-D-aspartate receptors (NMDAR) and L-type voltage-gated calcium channels (L-VGCC) during stimulation, and were not observed in the presence of cyclosporine A—an inhibitor of calcineurin protein phosphatase. Hence, rTMS may act on inhibitory synapses of principal neurons through Ca^2+^-dependent phosphorylation/dephosphorylation reactions which modulate the turnover/stability of gephyrin-scaffolds and thus the accumulation of GABA_A_ receptors at inhibitory postsynapses (Figure 1D, see also Zacchi et al., 2014).

Notably, rTMS-effects on gephyrin were also observed in the intact brain of anesthetized mice (Lenz et al., 2016). In light of the fact that rare exonic deletions implicate gephyrin in risk for autism, schizophrenia and seizures (e.g., Lionel et al., 2013), these findings make rTMS an interesting clinical tool for the modulation of gephyrin-mediated inhibitory synaptic plasticity. Together with previous work (Levkovitz et al., 1999; Benali et al., 2011), these experiments demonstrate that rTMS can lead to long-lasting changes in inhibitory neurotransmission. In case of 10 Hz stimulation rTMS induces LTD of inhibition, i.e., an *rTMS-induced disinhibition* of principal neurons.

## The Relationship between rTMS-Induced Disinhibition and Excitatory Synaptic Plasticity

Evidently the after-effects of rTMS consist of changes in inhibitory and excitatory synaptic strength. Since both synaptic effects required similar Ca^2+^-dependent signaling pathways, i.e., activation of NMDA-receptors and L-VGCC during stimulation (Huang et al., 2007; Vlachos et al., 2012; Labedi et al., 2014; Lenz et al., 2015, 2016), an important question that arises from these findings concerns the spatio-temporal interrelation between rTMS-induced LTD of inhibition and LTP of excitation.

The proposed model of rTMS-induced local disinhibition may suggest that rTMS acts primarily on GABAergic neurotransmission, while LTP of excitatory inputs rather reflects the outcome of ongoing network activity under conditions of rTMS-induced disinhibition. In other words: while 10 Hz rTMS induces robust LTD of inhibition, LTP of excitation may not be a direct effect of rTMS. This suggestion is also supported by basic animal research studies, which demonstrate that disinhibition promotes changes of specific excitatory synapses (Ormond and Woodin, 2009, 2011). Noteworthy, in our previous work (Vlachos et al., 2012) we did not observe a rapid post-tetanic-potentiation of excitatory synapses in response to 10 Hz magnetic stimulation, as seen in classic LTP experiments using local electrical stimulation (e.g., Strehl et al., 2014). Rather, a slow onset LTP is seen, which develops during the first 1–2 h and reaches a plateau 2–4 h after magnetic stimulation (Vlachos et al., 2012). Thus, LTP of excitation may not be an immediate consequence of 10 Hz magnetic stimulation. However, evidence has also been provided that inhibitory synapses are modified in response to LTP of excitatory synapses (Bannai et al., 2009). Accordingly, it will be important to now test for the precise temporal, spatial and molecular interrelation between rTMS induced LTD of inhibition (Lenz et al., 2016) and LTP of excitation (Vlachos et al., 2012; Lenz et al., 2015; see also Kozyrev et al., 2014).

It is quite attractive to put these considerations into the context of the lately discussed inter- and intra-individual variability of rTMS-induced after-effects in human subjects (Ziemann and Siebner, 2015). Hence, while the cellular and molecular mechanisms engaged by rTMS on cortical networks may not be that variable—given careful standardization—the outcome of rTMS-induced disinhibition could very well depend on the state of the stimulated network and ongoing activity. Based on this hypothesis the highly reproducible slow-onset LTP in our experimental setting may simply reflect the comparable (and low) network activity in our preparations across slice cultures and litters of different genotypes. Future basic research studies employing network activity modulation *before, during* and/or *after* rTMS will shed new important light on the relevance of rTMS-induced disinhibition and its role in promoting state-dependent homeostatic and metaplastic changes (see, Karabanov et al., 2015; Müller-Dahlhaus and Ziemann, 2015).

## Input-Specific Synaptic Effects of rTMS on Principal Neurons

**Figure 2:**
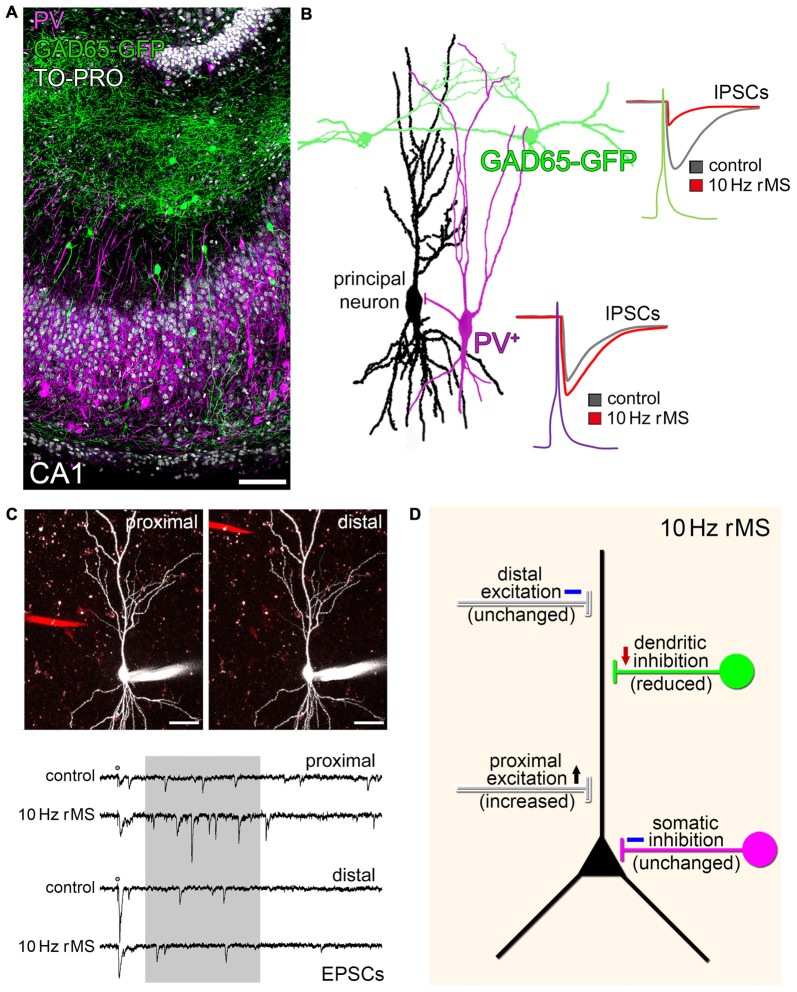
**rMS modulates network connectivity through changes of specific inputs on principal neurons. (A)** CA1 region in an organotypic slice culture prepared from glutamate decarboxylase (GAD65)-GFP mice stained for parvalbumin (PV; nuclear stain, TO-PRO^®^; scale bar: 100 μm). GFP identifies a population of interneurons that mainly project onto dendrites of CA1 pyramidal neurons, while PV-positive (PV^+^) interneurons mediate somatic inhibition. **(B)** Paired recordings disclose that 10 Hz rMS reduces dendritic inhibition, while not affecting somatic inhibition (inhibitory postsynaptic currents, IPSCs; drawing based on original data; see Lenz et al., 2016). **(C)** The same 10 Hz stimulation protocol increases excitatory synaptic strength on proximal, but not distal dendritic segments. An example of local electrical stimulation (stimulating electrode; red) while recording from the same CA1 pyramidal neuron, and corresponding sample traces of excitatory postsynaptic currents are shown (asynchronous EPSCs analyzed in a time range of 100–500 ms after stimulation (shaded area); scale bars: 40 μm; further experimental details provided in Lenz et al. (2015); copyright clearance obtained from Springer). **(D)** Schematic summarizing the major findings on input-specific effects of 10 Hz rMS in organotypic hippocampal slice cultures.

Regardless of the proposed model of rTMS-induced local disinhibition, which provides an attractive explanation of how a seemingly unspecific, i.e., exogenous electromagnetic stimulation could prime the ability of neurons to express endogenous, i.e., task- and input-specific excitatory synaptic plasticity, recent work has also demonstrated that rTMS *per se* may act through a selective modulation of specific inputs onto principal neurons (Vlachos et al., 2012; Lenz et al., 2015, 2016). Using immunostainings for gephyrin, GABA-uncaging experiments and paired-recordings of selected interneurons and pyramidal neurons, we were able to demonstrate that dendritic but not somatic inhibition is reduced in our experimental setting (Figures 2A,B; Lenz et al., 2016). In line with this observation the same 10 Hz stimulation protocol potentiates excitatory synapses predominantly on small spines of proximal dendrites on pyramidal neurons (Figure 2C; Vlachos et al., 2012; Lenz et al., 2015). These findings raise the intriguing possibility that rTMS may lead to input-specific synaptic changes, although an electromagnetic field is induced that covers a large volume of tissue. However, at this point we have to concede that we do not know enough about the mechanism and interplay between inhibitory and excitatory plasticity, which govern input-specific rTMS effects and their significance for brain function under physiological and pathological conditions (see discussion in Lenz et al., 2015, 2016).

Yet, from an anatomical point of view it is interesting to speculate that certain rTMS protocols may increase the efficacy of specific cortical inputs, e.g., layer-IV thalamo-cortical inputs onto proximal apical dendrites of layer-V pyramidal neurons, while gating the ability of the stimulated principal neurons to express task-specific associative synaptic plasticity, e.g., in the superficial associative layers, due to LTD-induction of dendritic inhibition (Figure 2D). Whether the input-selective effect of rTMS described in organotypic hippocampal slice cultures (Lenz et al., 2015, 2016) is a general phenomenon that is also detected in other cortical networks is a matter of current investigations.

## Some Open Questions and Future Directions

Apparently, 30 years after the development of the first TMS device by Barker et al. (1985), the cellular and molecular mechanisms of rTMS-induced neural plasticity have started to emerge (Tang et al., 2015). Despite its clinical use, however, rTMS-based therapies that prevent or reduce disabilities in a functionally significant and sustained manner remain scarce (Lefaucheur et al., 2014). Substantial progress in this field has been hampered by limitations in our understanding of the effects engaged by TMS in healthy and diseased brain tissue, and particularly its impact on excitatory and inhibitory synaptic plasticity. Another major limitation, which also prevents the efficient therapeutic application of rTMS, is the lack of a comprehensive understanding on the role of neural plasticity under pathological conditions.

Work from past years has indicated that synaptic plasticity may not only be altered or impaired under disease conditions, but dysregulated, i.e., maladaptive synaptic plasticity may initiate and even sustain the remodeling of neuronal networks and promote behavioral and cognitive deficits (see also Maggio and Vlachos, 2014). Our current understanding is that the threshold for synaptic plasticity events may change under pathological conditions. As discussed in this review article changes in excitation/inhibition-balance seem to play a crucial role in this context, since changes in inhibitory neurotransmission affect the ability of neurons for subsequent changes in excitatory synapses and vice versa under pathological conditions (Nahmani and Turrigiano, 2014). In addition, impairments of synaptic plasticity are not always detrimental, since it is possible that a reduction in the ability of neurons to express synaptic plasticity may protect neural networks from maladaptive changes. However, reduced plasticity may also hamper the functional recovery. Thus, a comprehensive understanding of the role of distinct forms of synaptic plasticity, i.e., associative plasticity, homeostatic plasticity and metaplasticity, under pathological conditions is urgently needed (Hulme et al., 2013; Maggio and Vlachos, 2014).

Future diagnostic and therapeutic approaches may aim at detecting, promoting, blocking or shifting the balance between various forms of plasticity in distinct brain regions at diverse stages of neurological diseases. Thereby, also overcoming or complementing major limitations of classic pharmacological therapeutic approaches, which lack sufficient spatio-temporal specificity and selectivity. Apparently, rTMS represents a promising tool in this context. While rTMS-induced changes in intrinsic cellular properties (Tang et al., 2016) and other mechanisms need to be considered, e.g., modulation of gene expression, mRNA-sorting and local protein synthesis, morphological changes, glial and vascular effects, we are confident that the emerging concept of *rTMS-induced local disinhibition* may provide an interesting framework for future studies.

In view of basic science work, it will be important for example to: (1) learn more about the cellular and molecular effects of rTMS on long-range excitatory and inhibitory projections onto local inhibitory interneurons; (2) better understand the effects of rTMS on glutamatergic and GABAergic neurotransmission onto specific subtypes of inhibitory interneurons; and (3) determine whether rTMS may also act through neuromodulators, which influence local inhibitory networks (see, Figure 1A). It will be also important to search for specific rTMS protocols that may increase inhibition and could thus hamper the ability of neurons to express LTP of excitatory synapses. A better understanding of the stimulation parameters which mediate input-specific synaptic changes, together with molecular studies on how rTMS modulates excitation/inhibition-balance, may help devising new therapeutic strategies using rTMS combined with pharmacological intervention. These studies could support for example, the development of more efficient rTMS-based therapies in post-stroke rehabilitation (Ziemann, 2005; Brodie et al., 2014; Smith and Stinear, 2016) or epilepsy (e.g., Carrette et al., 2016; Gersner et al., 2016), which already intend to target alterations in excitation/inhibition-balance. We hope *rTMS-induced local disinhibition* will be considered in future basic and clinical studies.

## Author Contributions

ML and AV prepared the figures and wrote this manuscript.

## Conflict of Interest Statement

The authors declare that the research was conducted in the absence of any commercial or financial relationships that could be construed as a potential conflict of interest.
